# Cardiovascular–kidney–metabolic syndrome: novel molecular mechanism and glucagon-like peptide-1-targeting therapy

**DOI:** 10.3389/fcell.2026.1996288

**Published:** 2026-10-23

**Authors:** Fengzhao Liu, Zihan Xu, Wenru Wang, Junwei Gao, Changgeng Fu, Peng Liu

**Affiliations:** 1 Xiyuan Hospital, China Academy of Chinese Medical Sciences, Beijing, China; 2 Ningbo Municipal Hospital of Traditional Chinese Medicine, Affiliated Hospital of Zhejiang Chinese Medical University, Ningbo, China

**Keywords:** cardiovascular disease, cardiovascular-kidney-metabolic syndrome, chronic kidney diseases, glucagon-like peptide-1, glucagon-like peptide-1 receptor agonists, natural product, obesity, type 2 diabetes

## Abstract

Cardiovascular–kidney–metabolic (CKM) syndrome represents a systemic disease continuum arising from the progressive and reciprocal interactions among metabolic dysfunction, kidney injury, and cardiovascular disease. Obesity and type 2 diabetes mellitus (T2DM) are major metabolic drivers of early CKM progression and contribute to cardiac and kidney injury through insulin resistance, glucotoxicity and lipotoxicity, chronic inflammation, oxidative stress, mitochondrial dysfunction, and ectopic lipid deposition, thereby establishing a self-reinforcing vicious cycle across the metabolic, cardiovascular, and renal systems. Glucagon-like peptide-1 (GLP-1) and its receptor (GLP-1R) not only participate in the regulation of glucose homeostasis and energy metabolism but also represent an important regulatory nexus linking metabolic dysfunction to cardiorenal injury. GLP-1 receptor agonists (GLP-1RAs) improve metabolic homeostasis by enhancing glucose-dependent insulin secretion, suppressing glucagon release, reducing appetite and body weight, and decreasing visceral and ectopic fat accumulation. In parallel, they may exert renal and cardiovascular protective effects by improving endothelial function and microcirculation, suppressing inflammation and oxidative stress, maintaining mitochondrial homeostasis, and attenuating fibrosis. In recent years, large-scale clinical studies have further extended the benefits of GLP-1RAs beyond glycaemic control and weight reduction to improved kidney outcomes, reduced major cardiovascular events, and benefits in obesity-related heart failure with preserved ejection fraction. Meanwhile, natural products, characterised by multi-component, multi-target, and multi-pathway actions, may provide a complementary strategy for integrated CKM management by simultaneously improving glucose and lipid metabolic disturbances, insulin resistance, inflammation, and oxidative stress, while attenuating cardiac remodelling, kidney injury, and fibrosis. Emerging studies have shown that some natural products can concurrently improve metabolic, cardiac, and renal phenotypes in CKM-related diseases or composite disease models. This review focuses on the interactions among the metabolic, renal, and cardiovascular systems in CKM, summarises the biological basis and signalling mechanisms of the GLP-1/GLP-1R system, and highlights the mechanistic and clinical evidence supporting GLP-1RA-mediated protection against obesity- and T2DM-related metabolic dysfunction and cardiorenal injury. The potential value of natural products in comprehensive CKM intervention and future research directions are also discussed.

## 1 Introduction

In 2023, the American Heart Association (AHA) introduced the term cardiovascular–kidney–metabolic syndrome (CKM) to describe a systemic disorder characterised by the interrelationships among obesity, type 2 diabetes mellitus (T2DM), chronic kidney disease (CKD), and cardiovascular disease (CVD) (Ndumele et al., 2023a; Khan S. S. et al., 2026). The AHA classifies CKM into stages 0–4 (Ndumele et al., 2026a; Levin et al., 2026). Stages 1 and 2 are characterised primarily by excess adiposity, T2DM, and other metabolic abnormalities (Claudel et al., 2025). A substantial body of evidence indicates that metabolic dysfunction drives the progression of CKM from early metabolic abnormalities to cardiac and kidney injury (Mavromatis and Grams, 2026; Li F. H. et al., 2026; Juguilon et al., 2025). Obesity and adipose tissue dysfunction disrupt glucose and lipid homeostasis through chronic inflammation, insulin resistance, and oxidative stress (Xu et al., 2026; Jankauskas et al., 2026). Persistent hyperglycaemia and dyslipidaemia further promote kidney and cardiovascular injury through glucotoxicity and lipotoxicity (Cai et al., 2025). The latest global estimates indicate that, in 2022, more than 1 billion people worldwide were living with obesity, including approximately 879 million adults and 159 million children and adolescents aged 5–19 years (Worldwide trends in underweight and, 2024; Minhas et al., 2024; Zhu et al., 2024). In 2024, an estimated 589 million adults aged 20–79 years were living with diabetes worldwide, corresponding to a global prevalence of 11.11% (Zhu et al., 2024; Xie et al., 2025). The high disease burden linked to obesity and diabetes indicates that CKM has developed into a significant public health concern that demands consideration (Xie et al., 2025; Genitsaridi et al.).

Currently, clinical management of CKM remains focused primarily on interventions targeting metabolic abnormalities, cardiovascular disease, or renal injury individually. Comprehensive prevention and treatment strategies addressing the full pathological continuum of CKM remain inadequate (Ndumele et al., 2023b; Rangaswami et al., 2026; Li Y. et al., 2026), partly owing to its complex multisystem pathogenesis. CKM is not merely a coexistence of metabolic, cardiovascular, and renal diseases; rather, it represents a disease continuum driven by interacting and self-amplifying pathological processes, including metabolic dysfunction, neurohumoral activation, oxidative stress, chronic inflammation, endothelial dysfunction, mitochondrial dysfunction, and fibrosis (Funahashi et al., 2025; Hatamizadeh, 2024; Zhao Z. et al., 2025; Rangaswami et al., 2019; Hatamizadeh et al., 2013; Zannad and Rossignol, 2018). Previous studies of cardiorenal syndrome have identified a bidirectional pathological link between the heart and kidneys involving renin–angiotensin–aldosterone system activation, inflammation, oxidative stress, dysregulated signalling, and profibrotic pathways. This crosstalk may also be modulated by gut microbiota dysbiosis and non-coding RNAs (Yang et al., 2025; Zhao et al., 2019; Chuppa et al., 2018). However, unlike cardio-renal syndrome, which focuses primarily on the interaction between the heart and kidneys, CKM places greater emphasis on metabolic disorders as upstream drivers. Obesity, adipose tissue dysfunction, insulin resistance, and T2DM increase cardiac and renal metabolic burden through glucotoxicity, lipotoxicity, and ectopic lipid deposition, thereby promoting oxidative stress, inflammation, mitochondrial dysfunction, and fibrosis. At the same time, kidney damage is not just a terminal manifestation of CKD affecting the target organs, but it may also act as a key factor in further progressing the disease. CKD, particularly diabetic kidney disease (DKD), is characterised by progressive structural injury, including extracellular matrix accumulation and tubular atrophy (De Gregorio et al., 2025; Liu Y., 2024). Its progression is closely associated with oxidative stress, inflammation (Liu H. et al., 2025; Wang et al., 2023a; Rayego-Mateos et al., 2023; Tang and Yiu, 2020), renin–angiotensin system dysregulation (Walther et al., 2021), and profibrotic signalling (Cao et al., 2022; Liu L. et al., 2026; Liu et al., 2026b), and may also involve disturbances in gut microbiota (Sun et al., 2026; Zhang W. Y. et al., 2026; Stepanova, 2024; He et al., 2025) and endogenous metabolites (Sharma et al., 2025; Li et al., 2024a; Corradi et al., 2024; Song et al., 2025; Liu et al., 2023; Li et al., 2024b).

Accordingly, within the CKM framework, the kidneys are both targets of metabolic dysfunction and active contributors to disease progression. Persistent inflammation, metabolic disturbance, and neurohumoral dysregulation arising from kidney injury may further aggravate cardiac and renal damage, creating a vicious cycle in which metabolic dysfunction and cardiorenal injury reinforce one another. These characteristics underscore the need to shift CKM prevention and treatment beyond single-risk-factor or single-organ interventions towards integrated strategies that address upstream metabolic abnormalities while simultaneously limiting downstream cardiac and renal injury.

GLP-1, an incretin hormone secreted by intestinal L cells (Daniels et al., 2025; Yabut and Drucker, 2026), stimulates glucose-dependent insulin secretion and suppresses glucagon release (Rosen and Ingelfinger, 2026; Stamouli et al., 2026). It also regulates glycaemia, appetite, and body weight (Wong et al., 2025; Li H. Y. et al., 2026). As understanding of the tissue distribution and biological actions of the GLP-1R has advanced, GLP-1RAs have been shown to extend beyond glucose lowering and weight reduction (Karacabeyli and Lacaille, 2025; Sabbagh et al., 2026; Mannucc et al., 2026), with potential effects on endothelial function, inflammation, and cardiorenal protection (Sabbagh et al., 2026; Ibe and Borlaug, 2026; Steven et al., 2025; Pugliese et al., 2025; Jalleh et al., 2026). These findings provide a biological rationale for their use in the comprehensive management of CKM (Lincoff et al., 2023; Tang et al., 2025; Dennis et al., 2026). Previous studies have largely examined the role of GLP-1 in obesity, T2DM, and their related complications (Lee et al., 2025a; Lee et al., 2025b; Alicic et al., 2026). However, research integrating its metabolic, cardiovascular, and renal effects within the CKM framework remains limited (Patel et al., 2026; Morissette et al., 2025). This review therefore focuses on early metabolic stress and dysfunction in CKM and considers how GLP-1/GLP-1R signalling may improve metabolic homeostasis and mitigate cardiac and renal injury. It aims to provide a theoretical basis for delaying CKM progression and optimising GLP-1R-targeted therapeutic strategies.

## 2 The biological basis of GLP-1 and GLP-1R

### 2.1 Secretion characteristics of GLP-1 and the structure and tissue distribution of the GLP-1 receptor

GLP-1 is a gastrointestinal hormone secreted primarily by intestinal L cells (Drucker, 2026). Its secretion increases after meals in response to nutrients, including glucose, fatty acids, and amino acids (Sodum et al., 2024). GLP-1R, a class B G protein-coupled receptor, comprises 463 amino acids and features a relatively long extracellular N-terminal domain, seven transmembrane helices, and an intracellular C-terminal domain (Zhou et al., 2025; Douros et al., 2024). GLP-1R is widely distributed across tissues (da Silva Lima et al., 2024). Its expression is not restricted to pancreatic islets but has also been reported in sinoatrial nodal myocytes, kidney vascular smooth muscle cells, multiple nuclei within the central nervous system, and selected cell populations in peripheral tissues (Pyke et al., 2014; Feng and Jin, 2025). This broad tissue distribution provides an anatomical basis for the cross-organ protective actions of GLP-1RAs. Beyond regulating glucose metabolism, GLP-1RAs may modulate CKM-related metabolic abnormalities, cardiovascular injury, and Kidney damage through endocrine and paracrine signalling across multiple organs.

### 2.2 Signal transduction mechanisms and physiological functions of GLP-1R

In pancreatic β-cells, GLP-1 binding stabilises the GLP-1R in its active conformation and promotes its coupling to the stimulatory G protein (Gs). This activates the Gsα–adenylate cyclase signalling axis, increasing intracellular cyclic adenosine monophosphate (cAMP) concentrations (Zheng Z. et al., 2024). Elevated cAMP amplifies glucose-stimulated insulin secretion through protein kinase A (PKA)-dependent signalling and a PKA-independent pathway mediated by exchange protein directly activated by cAMP 2 (Epac2) and Ras-related protein 1 (Rap1), constituting a central component of canonical GLP-1R signalling (Kashima et al., 2001; de Rooij et al., 1998). PKA phosphorylates transcriptional regulators, including cAMP-responsive element-binding protein (CREB), and thereby modulates transcription of the insulin gene and genes associated with β-cell survival, such as insulin receptor substrate 2. PKA also enhances glucose-stimulated insulin granule exocytosis by regulating ion channels, intracellular Ca^2+^ signalling, and the exocytotic machinery (Van de Velde et al., 2026; Liu Y. et al., 2026). Epac2 activates the small GTPase Rap1 and, through exocytosis-associated proteins such as Rab3-interacting molecule 2, regulates insulin granule recruitment, docking, priming, and exocytosis.

As noted above, GLP-1R is widely expressed across multiple tissues. Beyond glycaemic regulation, GLP-1/GLP-1R signalling contributes to lipid metabolism, renal function, and cardiovascular homeostasis, thereby linking metabolic regulation with target-organ function (Jones and Brierley, 2025). In adipose tissue, its effects on lipid metabolism are mediated predominantly through indirect mechanisms, including central and autonomic nervous system regulation and improved systemic metabolism. These actions reduce energy intake, improve insulin sensitivity, promote lipid utilisation, and ultimately limit visceral and ectopic lipid accumulation (Jones and Brierley, 2025). GLP-1R signalling extends beyond the canonical Gs–cAMP–PKA/Epac2 axis. As a class B G protein-coupled receptor, GLP-1R can adopt distinct conformations that generate signalling bias, recruit β-arrestin, regulate mitogen-activated protein kinase/extracellular signal-regulated kinase (MAPK/ERK) signalling, and engage non-canonical pathways, including phospholipase C (PLC)–inositol 1,4,5-trisphosphate (IP3)/Ca^2+^/protein kinase C (PKC) and phosphoinositide 3-kinase (PI3K)/Akt (Douros et al., 2024; Liu L. et al., 2025). The diverse tissue-specific actions of GLP-1R may reflect differences in signalling intensity, duration, and receptor endocytosis across these pathways (Douros et al., 2024). In cardiac and vascular tissues, β-arrestin–PI3K/Akt and PI3K/Akt–endothelial nitric oxide synthase (eNOS)/nitric oxide (NO) signalling have been linked to mitochondrial quality control, cell survival, and endothelial protection. Under hyperglycaemic conditions, reduced β-arrestin expression attenuated GLP-1-mediated protection against hypoxia/reoxygenation injury in cardiomyocytes. Restoration of β-arrestin signalling improved mitochondrial quality control through PI3K/Akt activation and reinstated the cardioprotective effects of GLP-1 (Pan et al., 2021). Furthermore, GLP-1RAs may modulate endothelial function, oxidative stress, and NO production through phosphoinositide 3-kinase/Akt-dependent downstream signalling (Wang T. et al., 2024; Winquist and Gribkoff, 2022). In cardiac microvascular endothelial cells, liraglutide attenuated Ca^2+^ overload and oxidative injury through the GLP-1R/PI3K/Akt/survivin pathway, thereby preserving intracellular Ca^2+^ homeostasis (Zhang et al., 2016). These effects may provide a molecular basis for GLP-1RAs to improve myocardial metabolism, microcirculation, and oxidative stress.

In the kidney, GLP-1R signalling contributes to the regulation of renal haemodynamics and sodium homeostasis. GLP-1 inhibits proximal tubular sodium reabsorption and promotes natriuresis through cAMP/PKA -dependent phosphorylation of sodium–hydrogen exchanger 3 (Caruso and Giorgino, 2024; Rojano Toimil and Ciudin, 2021). Beyond these haemodynamic actions, GLP-1 and GLP-1RAs may modulate signalling networks involved in renal metabolism and cellular injury. For example, GLP-1-related enhanced silent information regulator 1 (SIRT1)/AMP-activated protein kinase (AMPK)/peroxisome proliferator-activated receptor γ coactivator 1α (PGC-1α) signalling has been associated with reduced renal lipid accumulation, improved mitochondrial function, and attenuated fibrosis (Han et al., 2024). Liraglutide has also been reported to alleviate oxidative stress and extracellular matrix deposition by promoting nuclear translocation of nuclear factor erythroid 2-related factor 2 (Nrf2) (Lin T. et al., 2024). In addition, GLP-1R signalling may suppress receptor for advanced glycation end products (RAGEs)-associated inflammatory responses (Sourris et al., 2024). Collectively, these findings suggest that GLP-1RAs may exert renoprotective effects through coordinated modulation of renal haemodynamics, metabolism, oxidative stress, and inflammation (Figure 1).

**FIGURE 1:**
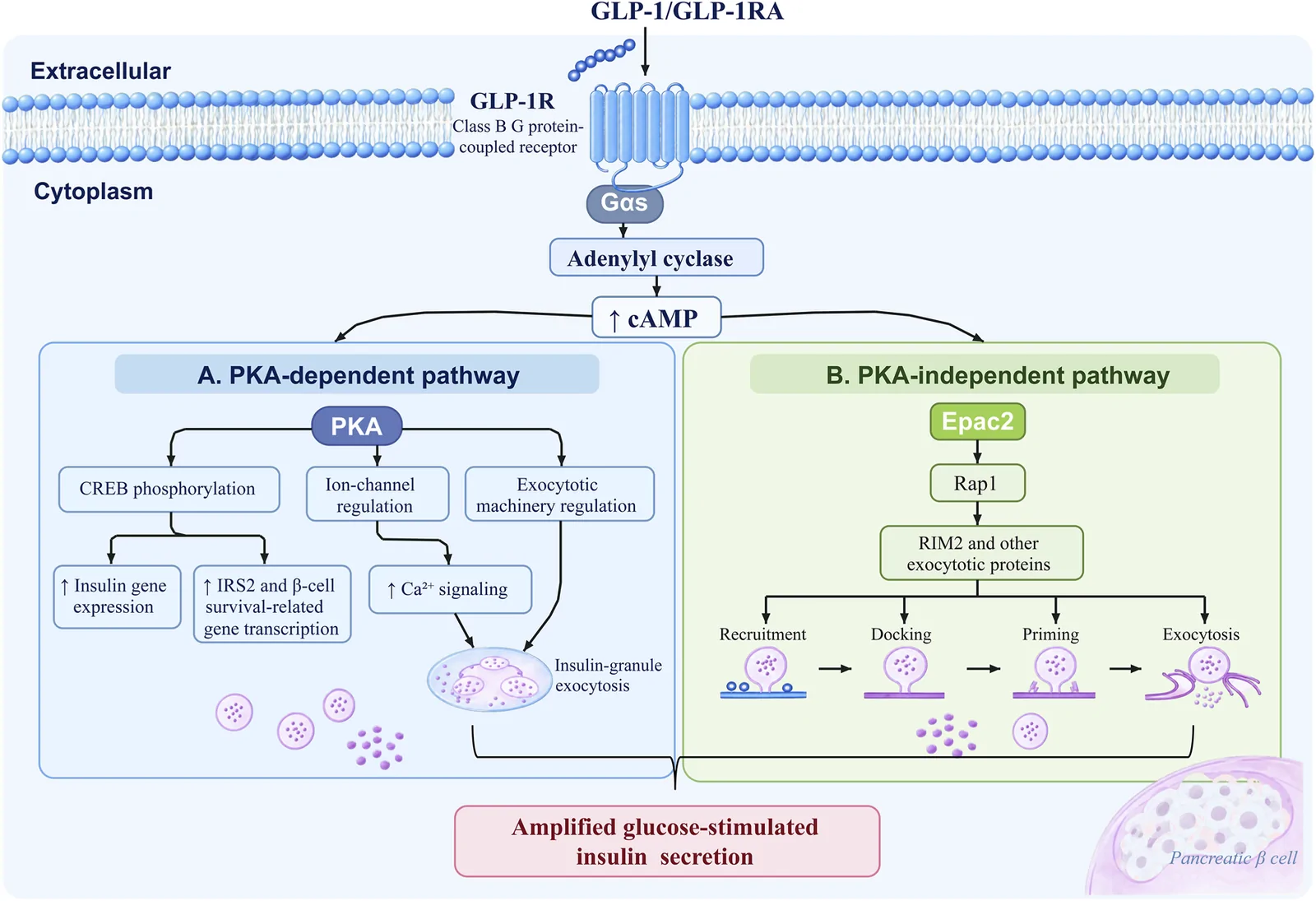
Canonical GLP-1R Signaling Pathways Mediating Glucose-Stimulated Insulin Secretion in Pancreatic β-Cells. Binding of GLP-1 or GLP-1RAs to GLP-1R activates Gαs and adenylyl cyclase, leading to increased intracellular cAMP. Elevated cAMP promotes insulin secretion through both PKA-dependent and PKA-independent pathways. PKA activation regulates CREB phosphorylation, ion-channel activity, Ca^2+^signaling, and the exocytotic machinery, thereby enhancing insulin gene expression, β-cell survival-related transcription, and insulin-granule exocytosis. In parallel, cAMP activates Epac2–Rap1 signaling, which regulates RIM2 and other exocytotic proteins to facilitate insulin-granule recruitment, docking, priming, and exocytosis. Together, these pathways amplify glucose-stimulated insulin secretion in pancreatic β cells.

## 3 CKM-related metabolic abnormalities and cardiac and kidney damage

### 3.1 Obesity-related cardiac and kidney damage

Adipose tissue dysfunction, together with overweight and obesity, is a major driver of CKM onset and progression (Wong et al., 2025; Drucker, 2025). This dysfunction and the ensuing ectopic lipid deposition are closely associated with cardiac and kidney injury (Jankauskas et al., 2026; Khanna et al., 2026). Lipotoxicity arises when adipose tissue can no longer adequately store excess lipids, resulting in their transport to and ectopic deposition within non-adipose tissues (Liu Z. et al., 2025). This process is accompanied by the accumulation of bioactive lipid intermediates, including ceramides and diacylglycerols (Carobbio et al., 2024). Excessive adipose tissue expansion promotes adipocyte hypertrophy, hypoxia, oxidative stress, inflammation, and secretory dysfunction. Consequently, the release of free fatty acids, leptin, tumour necrosis factor-α (TNF-α), interleukin-6, and monocyte chemoattractant protein-1 increases, whereas adiponectin concentrations decline, promoting insulin resistance and chronic low-grade systemic inflammation (Li et al., 2023a). As adipose tissue storage capacity becomes saturated, lipid overflow promotes ectopic deposition in the myocardium, liver, and kidneys, accompanied by expansion of epicardial and kidney sinus adipose tissue and the development of lipotoxicity. Concurrently, insulin resistance and secondary hyperglycaemia may exacerbate glucotoxicity, jointly driving CKM progression from early adipose tissue dysfunction to metabolic risk-factor accumulation and cardiokidney injury (Ahmed et al., 2025). Therefore, ameliorating obesity and adipose tissue dysfunction is essential for delaying CKM progression and provides a pathophysiological rationale for the cardioprotective and renoprotective effects of GLP-1RAs through metabolic regulation.

#### 3.1.1 Obesity-related kidney damage

In obesity, enhanced tubular sodium reabsorption reduces NaCl delivery to the macula densa, thereby attenuating tubuloglomerular feedback and decreasing afferent arteriolar resistance. Concurrent obesity-related activation of the renin–angiotensin–aldosterone system (RAAS) increases efferent arteriolar resistance. The combination of afferent arteriolar vasodilation and efferent arteriolar vasoconstriction elevates kidney blood flow, glomerular filtration rate, and intraglomerular pressure (Furth et al., 2026). Consequently, glomerular hyperfiltration may occur during the early stages of CKM, presenting as a normal or elevated estimated glomerular filtration rate (eGFR) (Lee-Boey et al., 2025). Persistently elevated glomerular filtration can induce glomerular hypertrophy and increase mechanical stress on podocytes, promoting podocyte injury and detachment and ultimately leading to proteinuria, focal segmental glomerulosclerosis, and kidney dysfunction (Kanbay et al., 2024). Minakawa et al. reported that, in 6-week-old obese Zucker rats—before the onset of hyperglycaemia and albuminuria—glomerular volume was already 1.6-fold greater than in controls. This was accompanied by podocyte hypertrophy, activation of the mammalian target of rapamycin complex 1 signalling pathway, and increased podocyte detachment. As disease progressed, reduced podocyte density was closely associated with albuminuria; by 46 weeks of age, approximately 47% of glomeruli exhibited segmental sclerosis (Minakawa et al., 2019). Collectively, these clinical and experimental findings suggest that obesity-related kidney injury may precede detectable abnormalities in routine kidney function measures and represent a key mechanism driving the transition from early CKM to structural kidney injury. At the cellular level, free fatty acids and cholesterol may accumulate aberrantly in podocytes, mesangial cells, and proximal tubular epithelial cells. When fatty acid uptake exceeds the capacity for β-oxidation and intracellular lipid storage, mitochondrial metabolic stress increases, ATP production declines, and reactive oxygen species (ROS) accumulation and endoplasmic reticulum stress ensue (Mitrofanova et al., 2023). This lipid overload causes cell type-specific injury: in podocytes, it disrupts slit diaphragm integrity and the glomerular filtration barrier, thereby promoting proteinuria; in tubular epithelial cells, it activates inflammasomes and cell-death pathways, exacerbating tubular injury and interstitial fibrosis (Ducasa et al., 2019). Concurrently, oxidised low-density lipoprotein, once taken up by mesangial cells and infiltrating macrophages, promotes foam-cell formation and local inflammation while exacerbating mesangial matrix deposition and glomerulosclerosis. kidney lipid accumulation can therefore drive the transition from early cellular dysfunction to structural kidney injury through metabolic stress, inflammation, and cellular damage.

#### 3.1.2 Obesity-related cardiac damage

In the cardiovascular system, obesity increases circulating blood volume, venous return, and cardiac output, exposing the heart to chronically elevated preload. Simultaneously, obesity-related activation of the sympathetic nervous system and RAAS, together with hypertension and arterial stiffness, increases cardiac afterload (Savarese et al., 2025). Sustained volume and pressure overload may lead to left ventricular hypertrophy, left atrial enlargement, myocardial interstitial fibrosis, and diastolic dysfunction (Borlaug et al., 2023a; Borlaug et al., 2023b). Obesity is also closely associated with an increased risk of heart failure (HF), particularly HF with preserved ejection fraction (HFpEF). In the Framingham Heart Study, which included 5,881 adults, each 1 kg/m^2^ increase in body mass index (BMI) was associated with a 5% higher risk of HF in men and a 7% higher risk in women. Compared with individuals of normal weight, obese men and women had 1.90-fold and 2.12-fold higher risks of HF, respectively (Kenchaiah et al., 2002). These findings indicate a graded association between increasing BMI and HF risk.

At the cellular level, cardiomyocytes normally retain metabolic flexibility, switching among fatty acids, glucose, and lactate according to substrate availability; fatty acids generally constitute their principal energy source (Goldberg et al., 2026). Obesity and insulin resistance, however, promote sustained fatty acid delivery and uptake, thereby impairing myocardial metabolic flexibility. When fatty acid influx exceeds the capacity of cardiomyocyte mitochondria for oxidation and safe lipid storage, bioactive intermediates, including ceramides and diacylglycerols, accumulate. These changes induce mitochondrial dysfunction, oxidative stress, disturbed calcium homeostasis, cardiomyocyte apoptosis, and fibrosis, thereby promoting cardiac steatosis and lipotoxic cardiomyopathy (Wilkerson et al., 2024).

### 3.2 T2DM-related glucolipotoxicity and cardiorenal injury

T2DM is a major metabolic abnormality driving CKM progression. A diagnosis of T2DM generally indicates that an individual has reached at least stage 2 of CKM. Glucotoxicity is a key mechanism through which disordered glucose metabolism causes cardiokidney injury in CKM and refers to cellular and tissue damage resulting from persistent or recurrent hyperglycaemic exposure. Hyperglycaemia promotes the formation of AGEs and activates their receptor (RAGE), while enhancing flux through the polyol and hexosamine pathways, increasing diacylglycerol accumulation, and activating PKC (González et al., 2023). These changes increase mitochondrial ROS production and activate nuclear factor-κB (NF-κB) and transforming growth factor-β (TGF-β) signalling, leading to inflammation, endothelial dysfunction, extracellular matrix deposition, and fibrosis. Together, these processes cause structural and functional cardiac and kidney injury and drive CKM progression from the metabolic risk-factor stage to subclinical and clinical cardiokidney disease (Fan et al., 2024; Liu et al., 2024a). Beyond structural cellular injury, single-cell studies have revealed marked functional heterogeneity among immune cells in DKD, together with active intercellular communication between kidney tubular epithelial cells and immune populations. These findings suggest that remodelling of the local immune microenvironment is an important component of inflammatory progression in DKD (Bai et al., 2024). Cellular injury in DKD is also not attributable to a single mode of cell death. Multiple regulated cell death pathways, including apoptosis, ferroptosis, pyroptosis and necroptosis, may coexist and interact. Together with dysregulated autophagic homeostasis, these processes contribute to podocyte and kidney tubular epithelial cell injury and thereby promote DKD progression (Liu et al., 2024b).

#### 3.2.1 T2DM-related kidney damage

T2DM-related kidney injury evolves progressively, from functional disturbances in multiple kidney parenchymal cell types to glomerular, tubulointerstitial, and kidney vascular remodelling, resulting in heterogeneous clinical phenotypes (Hu et al., 2024). DKD is a common microvascular complication of T2DM and a form of CKD. Early DKD is generally asymptomatic, although some patients develop glomerular hyperfiltration followed by a gradual increase in urinary albumin excretion. In a 4.0-year follow-up study of 600 patients with T2DM and hypertension who had normal or mildly elevated urinary albumin excretion, Ruggenenti et al. identified glomerular hyperfiltration at baseline in 90 participants. Among patients with persistent hyperfiltration, 23.4% progressed to microalbuminuria or macroalbuminuria, compared with 10.6% of those whose hyperfiltration resolved or who did not have hyperfiltration at baseline (Ruggenenti et al., 2012). These findings suggest that glomerular hyperfiltration may represent an early manifestation of DKD progression rather than merely a compensatory response. The classical course of DKD involves progression from mild to severe albuminuria, accompanied by a sustained decline in eGFR. However, DKD follows heterogeneous trajectories: some patients have persistently normal or mildly increased albuminuria, with eGFR decline as the predominant feature, resulting in non-albuminuric DKD (Shi et al., 2022). As kidney injury advances, hypertension, fluid and sodium retention, electrolyte disturbances, and anaemia may develop, and some patients ultimately progress to kidney failure. Reducing hyperglycaemic burden and preserving kidney function are therefore important components of comprehensive CKM management with GLP-1RAs.

At the cellular level, persistent hyperglycaemia, insulin resistance, and metabolic substrate excess synergistically injure glomerular endothelial cells, mesangial cells, podocytes, and tubular epithelial cells by inducing oxidative stress, inflammation, and profibrotic signalling (Dwivedi and Sikarwar, 2025). Glomerular endothelial injury is characterised primarily by increased ROS production and reduced nitric oxide bioavailability, resulting in barrier dysfunction and increased albumin permeability (Yu H. et al., 2023). In parallel, AGEs, PKC, and TGF-β signalling promote mesangial-cell activation and extracellular matrix synthesis, leading to mesangial expansion. Endothelial injury and mesangial expansion disrupt the glomerular microenvironment, inducing podocyte cytoskeletal remodelling, foot process effacement, and abnormalities in slit diaphragm proteins. These changes can cause podocyte hypertrophy, detachment, or death, ultimately disrupting the glomerular filtration barrier and promoting proteinuria and glomerulosclerosis (Li et al., 2023b). Beyond structural injury, single-cell studies have revealed marked functional heterogeneity among immune cells in DKD kidneys, together with extensive intercellular communication between immune cells and renal tubular epithelial cells. These findings implicate remodelling of the local immune microenvironment in the propagation of renal inflammation in DKD (Bai et al., 2024). Moreover, cellular injury is not attributable to a single mode of cell death. Multiple regulated cell death pathways, including apoptosis, ferroptosis, pyroptosis, and necroptosis, may coexist and interact. Alongside dysregulated autophagic homeostasis, these processes contribute to podocyte and tubular epithelial cell injury and may thereby promote DKD progression. Specifically, Li et al. found that DPEP1 expression was significantly increased in renal tubular epithelial cells from patients with DKD and in mouse models, and correlated with the severity of tubular injury and ferroptosis. In high-glucose-treated HK-2 cells, DPEP1 promoted ferroptosis by suppressing the GSH/GPX4 antioxidant axis, whereas DPEP1 knockdown or pharmacological inhibition attenuated ferroptosis and renal injury (Li et al., 2024c). Furthermore, the IRE1α/sXBP1 signalling pathway, NLRP3 inflammasome, and pyroptosis are markedly activated in the renal tubules of patients with DKD. Mechanistically, high-glucose exposure promotes NLRP3-mediated pyroptosis through IRE1α/sXBP1 signalling, whereas inhibition of this pathway attenuates tubular inflammation and injury in streptozotocin (STZ)-induced and db/db mouse models (Liu X. et al., 2025). Similarly, high-glucose exposure can induce necroptosis in renal tubular epithelial cells through ROS-dependent RIPK1/RIPK3/MLKL signalling. Pharmacological inhibition of RIPK1 or suppression of ROS production attenuates inflammatory responses and renal injury in diabetic mice (Guo et al., 2023). Collectively, these findings suggest that multiple forms of regulated cell death may act in concert under sustained hyperglycaemic and metabolic stress, contributing to persistent tubular injury and DKD progression.

#### 3.2.2 T2DM-related cardiac damage

Early cardiac injury associated with T2DM is often clinically silent. In some patients, the only manifestations are reduced exercise tolerance, abnormal myocardial strain, or impaired left ventricular diastolic function. Diabetic cardiomyopathy denotes structural and functional myocardial abnormalities occurring in the absence of coronary artery disease, hypertension, or significant valvular heart disease. It commonly presents initially as HFpEF, with systolic dysfunction emerging as the condition progresses. The DIABET-IC prospective cohort study enrolled 1,517 patients with T2DM from 58 centres in Spain and followed them for 3 years. Among incident HF cases, 46.6% had HFpEF, while 4.7% of patients with pre-existing HFpEF progressed HF with reduced ejection fraction (HFrEF). T2DM may also accelerate coronary atherosclerosis and increase the risk of myocardial infarction, HF, and atrial fibrillation. In a Swedish nationwide cohort study involving 617,000 patients with T2DM and more than 2.3 million matched controls, followed for a median of 7.6 years, T2DM was associated with an approximately 30% higher risk of atrial fibrillation or atrial flutter (Rawshani et al., 2023). Beyond long-term glycaemic exposure, glycaemic variability itself may contribute to cardiovascular risk. In a study of 484 patients with T2DM and HF following acute ST-segment elevation myocardial infarction, greater glycaemic variability at admission was associated with a higher risk of major adverse cardiovascular events within 1 year and showed greater predictive value than concurrently measured HbA1c (Yang X. et al., 2024).

At the cellular level, insulin resistance in cardiomyocytes reduces glucose uptake and shifts myocardial substrate use from glucose oxidation towards fatty acid oxidation (Heather et al., 2024). When fatty acid supply exceeds mitochondrial oxidative capacity, lipid intermediates, including diacylglycerols, ceramides, and acylcarnitines, accumulate, inducing oxidative stress and mitochondrial dysfunction. These changes reduce ATP-generating efficiency and myocardial energy reserves, thereby impairing contractile function (Actis Dato et al., 2024). Beyond their direct effects on cardiomyocytes, persistent hyperglycaemia causes coronary endothelial dysfunction, promotes low-density lipoprotein glycation, inflammatory-cell recruitment, and platelet activation, and consequently accelerates coronary atherosclerosis and atherothrombosis (Batten et al., 2023). Recent studies have further implicated regulated cell death in T2DM-associated myocardial injury. Wu et al. found aberrant activation of USP24/NF-κB signalling in mice with high-fat diet (HFD) - and STZ -induced diabetic cardiomyopathy and in cardiomyocytes exposed to high glucose and palmitic acid. This activation was accompanied by iron accumulation, increased FACL4 expression, and reduced SLC7A11 and FTH1 expression. Inhibition of USP24 or NF-κB attenuated these alterations, supporting the involvement of ferroptosis in diabetic myocardial injury (Wu et al., 2024). Furthermore, Wang et al. found that activation of the NLRP3 inflammasome in diabetic myocardium was accompanied by enhanced GSDMD -associated pyroptosis and abnormal xCT/GPX4-associated ferroptosis. Inhibiting the NLRP3 inflammasome attenuated both forms of cell death and myocardial injury. However, further increasing mtROS reversed this protective effect, suggesting that mtROS–NLRP3 may be a key link between metabolic stress and cardiomyocyte death (Wang J. et al., 2024). Together, these results imply that, alongside conventional oxidative stress and mitochondrial dysfunction, programmed cell death pathways such as pyroptosis and ferroptosis may contribute to T2DM-related myocardial remodelling and functional impairment (Figure 2).

**FIGURE 2:**
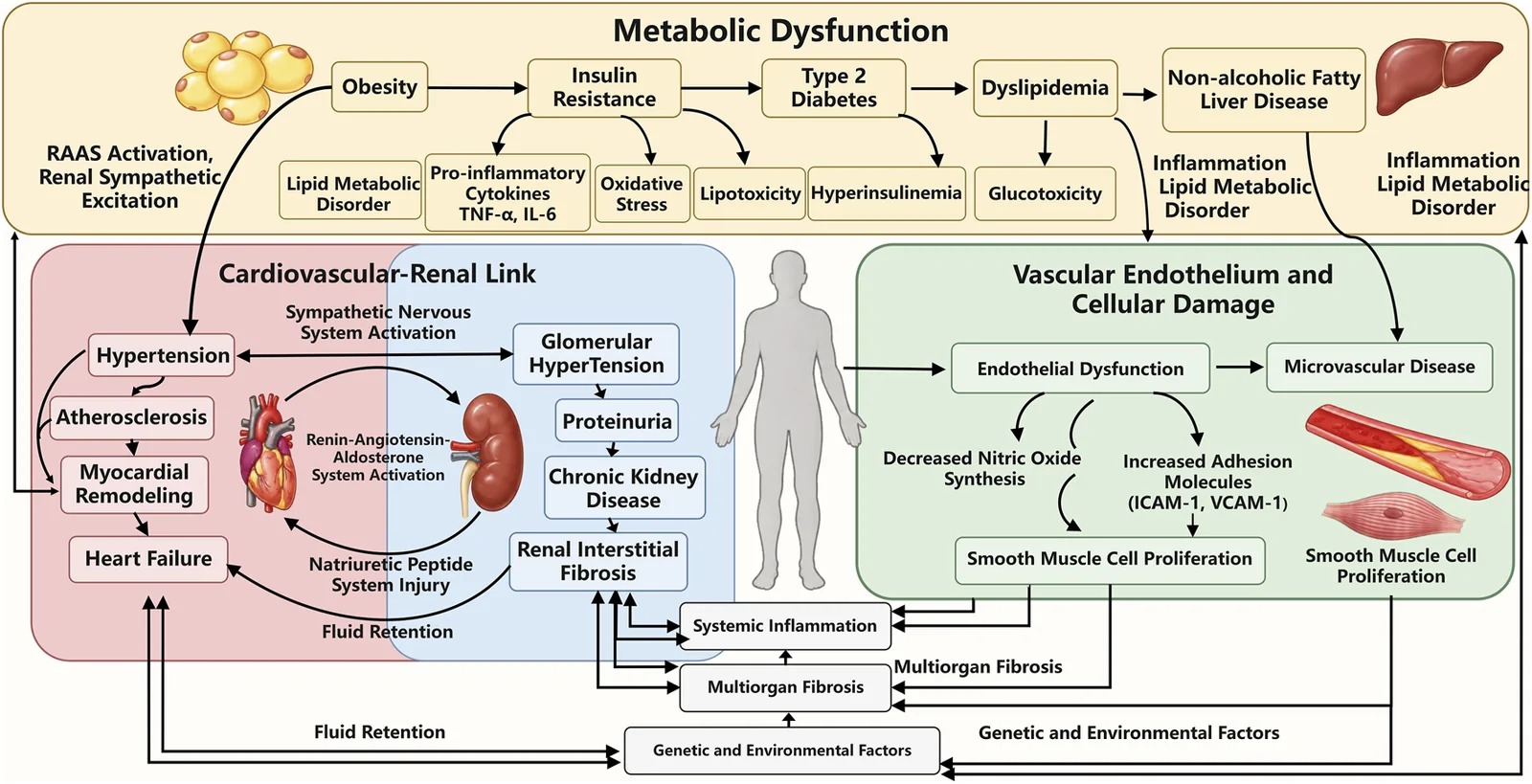
Pathophysiological mechanisms underlying CKM syndrome. Metabolic dysfunction, including obesity, insulin resistance, type 2 diabetes mellitus, dyslipidaemia, and abnormal lipid accumulation, represents a major upstream driver of CKM syndrome. These metabolic abnormalities promote glucotoxicity, lipotoxicity, chronic inflammation, oxidative stress, and hyperinsulinaemia, thereby contributing to endothelial dysfunction, microvascular injury, and progressive cardiovascular and kidney damage. Cardiovascular and kidney dysfunction further interact bidirectionally through haemodynamic disturbances, activation of the renin–angiotensin system and sympathetic nervous system, and fluid retention, promoting hypertension, atherosclerosis, cardiac remodelling, heart failure, albuminuria, chronic kidney disease, and renal fibrosis. Systemic inflammation, multiorgan fibrosis, and genetic and environmental factors further reinforce these interconnected processes, establishing a self-perpetuating cycle that drives CKM progression.

#### 3.2.3 Glucolipotoxic interaction in kidney and cardiac injury

As CKM progresses from stage 1, characterised primarily by excess or dysfunctional adipose tissue, to stage 2, marked by T2DM and other metabolic risk factors, glucotoxicity and lipotoxicity interact to create a self-reinforcing metabolic cycle. Obesity-related adipose tissue dysfunction and insulin resistance promote lipolysis, increasing circulating free fatty acid availability and lipid deposition in non-adipose tissues, including the kidneys and heart. As insulin resistance worsens and persistent hyperglycaemia develops, fatty acid uptake and lipid synthesis increase, whereas fatty acid oxidation and lipid clearance decline. These changes promote the accumulation of triglycerides and bioactive lipotoxic intermediates, including diacylglycerols and ceramides. Conversely, lipid overload and lipotoxic intermediates can impair insulin signalling and mitochondrial energy metabolism, increase ROS production, and further exacerbate insulin resistance and dysregulated glucose metabolism. Thus, obesity-related lipotoxicity and T2DM-related glucotoxicity not only coexist during early CKM progression but also mutually amplify one another through shared mechanisms, including insulin resistance, oxidative stress, mitochondrial dysfunction, endoplasmic reticulum stress, and chronic inflammation, thereby establishing a self-reinforcing glucotoxic–lipotoxic network. At the cellular level, persistent glucolipotoxic stress disrupts cellular homeostasis and can trigger multiple forms of regulated cell death, including apoptosis, pyroptosis, ferroptosis, and necroptosis. These processes promote the transition from metabolic dysfunction to structural and functional injury in the kidneys and heart.

The kidneys, particularly proximal tubular epithelial cells, are highly susceptible to this combined glucose–lipid metabolic burden because of their substantial reliance on mitochondrial fatty acid oxidation for energy. In obesity complicated by type 2 diabetes mellitus (T2DM), sustained hyperglycaemia and fatty acid overload can jointly increase tubular lipid uptake and synthesis while inhibiting fatty acid oxidation and clearance. This promotes ectopic lipid deposition, ATP deficiency, mitochondrial ROS generation, and endoplasmic reticulum stress. Clinical studies provide supportive evidence for this process. A quantitative multi-organ MRI study reported a high prevalence of renal fat accumulation and fibro-inflammatory changes in individuals with T2DM and identified obesity as closely associated with renal fat deposition and multi-organ abnormalities (Diam et al., 2024). Clinical and pathological studies have likewise demonstrated marked lipid-droplet accumulation in renal tubules of patients with DKD, which correlates with impaired renal function and histological injury (Yu Q. et al., 2023). Sustained glucolipotoxic stress may further aggravate tubular injury by triggering multiple forms of regulated cell death. Yu et al. reported increased RIPK1/RIPK3/MLKL-mediated necroptosis in renal tubules from patients with DKD. Phosphorylated MLKL (p-MLKL), a key executor of necroptosis, was positively correlated with renal functional impairment, pathological severity, and lipid-droplet accumulation (Yu Q. et al., 2023). High-fat feeding further exacerbated renal injury and activated necroptosis in db/db mice. In HK-2 cells, p-MLKL membrane translocation was observed predominantly after combined high-glucose and palmitate exposure, further supporting a link between glucolipotoxic stress and tubular necroptosis (Yu Q. et al., 2023). Khan et al. found that a HFD combined with STZ-induced severe hyperglycaemia caused more pronounced renal dysfunction, endoplasmic reticulum stress, NLRP3/caspase-1-mediated pyroptosis, and fibrosis than STZ-induced severe hyperglycaemia alone. *In vitro*, combined high-glucose and palmitate exposure activated Gα12–IRE1-associated endoplasmic reticulum stress and more strongly promoted pyroptotic and profibrotic responses than high glucose alone (Khan M. S. et al., 2026). Moreover, human renal tubular epithelial cells exposed to high glucose plus palmitate showed increased iron accumulation, ROS, and lipid peroxidation, accompanied by impairment of anti-ferroptotic defences, including GPX4 and system Xc^−^. These findings suggest that ferroptosis may also contribute to glucolipotoxic tubular injury (Zhu et al., 2025).

Similar glucose–lipid interactions occur in the myocardium. Insulin resistance in T2DM reduces myocardial glucose uptake and oxidation, while obesity-associated adipose tissue dysfunction increases circulating free fatty acid delivery to the heart, further shifting substrate utilisation towards fatty acids. When fatty acid uptake exceeds mitochondrial oxidative and safe-storage capacity, bioactive lipid intermediates—including diacylglycerols, ceramides, and lipid peroxidation products—accumulate and promote cardiomyocyte injury through oxidative stress and mitochondrial dysfunction. Clinical studies also suggest that coexisting obesity and T2DM exacerbate myocardial injury: compared with non-obese individuals with T2DM, those with obesity exhibit higher myocardial extracellular volume fraction and more pronounced subclinical left-ventricular systolic dysfunction (Li et al., 2025). Cardiac magnetic resonance imaging studies have shown that, among individuals without overt cardiovascular disease, those with both overweight and T2DM have greater visceral adiposity and are the only group to exhibit significantly reduced stress myocardial blood flow, accompanied by lower left ventricular ejection fraction and impaired global longitudinal strain. These findings suggest that obesity may exacerbate T2DM-associated coronary microvascular and myocardial dysfunction (Chowdhary et al., 2022). Experimental evidence further supports the contribution of combined glucose–lipid metabolic stress to myocardial injury. Moharamzadeh et al. compared HFD-induced obese mice with HFD + STZ-induced diabetic mice and found that HFD alone caused predominantly mild myocardial degenerative changes, whereas HFD + STZ induced more pronounced myocardial steatosis, fibrosis, and degenerative injury (Moharamzadeh et al., 2025). Karatzia et al. directly compared six modelling strategies involving HFD, STZ, and HFD + STZ. HFD alone predominantly induced diastolic dysfunction, whereas HFD + STZ produced a more comprehensive diabetic cardiomyopathy phenotype. This effect was particularly evident in female mice, which developed concurrent systolic and diastolic dysfunction, reduced myocardial energy reserves, and extensive metabolic remodelling (Karatzia et al., 2025). Buchanan et al. compared obese ob/ob mice with obese diabetic db/db mice and found that both models displayed increased myocardial fatty acid oxidation at early stages. However, persistent hyperglycaemia in db/db mice further increased fatty acid oxidation and was accompanied by progressive systolic and diastolic dysfunction, whereas ob/ob mice maintained high myocardial contractility. These findings suggest that sustained hyperglycaemia may exacerbate obesity-related disturbances in myocardial substrate utilisation and cardiac function (Buchanan et al., 2005).

## 4 Pharmacological basis and clinical evidence for GLP-1RA intervention in CKM

### 4.1 The effects of GLP-1 receptor agonists on obesity-related CKM

#### 4.1.1 Total body fat mass and ectopic lipid deposition

GLP-1RAs may mitigate lipotoxicity by reducing lipid availability, improving adipose tissue distribution, and enhancing tissue lipid handling (Rivera et al., 2025). By suppressing appetite and reducing energy intake, GLP-1RAs decrease body weight and visceral adiposity. They may also lower circulating free fatty acid concentrations and limit fatty acid delivery to non-adipose tissues, including the heart and kidneys, by improving insulin sensitivity and suppressing excessive adipose tissue lipolysis. In addition, GLP-1RAs reduce intestinal apolipoprotein B48 production, improve postprandial hyperlipidaemia, and lower remnant lipoprotein concentrations. Hoffman et al. showed that, in hamsters and mice, portal-vein administration of GLP-1 or activation of GLP-1Rs in the nodose ganglion reduced postprandial plasma triglyceride and triglyceride-rich lipoprotein concentrations. Conversely, vagal afferent blockade, sympathetic blockade, or GLP-1R knockout attenuated these effects (Hoffman et al., 2022). Regarding adipose distribution, a 2023 meta-analysis by Akoumianakis et al., which included 35 clinical studies, showed that GLP-1 analogues reduce visceral, subcutaneous, epicardial, and hepatic fat (Akoumian et al., 2023). These findings indicate that GLP-1RAs not only reduce total fat mass but also improve metabolically harmful visceral and ectopic fat deposition, thereby alleviating obesity-related metabolic disturbances. Compared with weight loss alone, reductions in visceral adiposity and ectopic lipid burden may more directly reflect the therapeutic value of GLP-1RAs in CKM.

Basic studies have further elucidated mechanisms through which GLP-1RAs may enhance tissue lipid handling. In obesity, uncontrolled adipose tissue lipolysis elevates circulating free fatty acid concentrations, whereas intracellular lipid mobilisation coupled with fatty acid oxidation facilitates the clearance of excess lipid stores. Xu et al. reported that exenatide reduced fat mass, adipocyte triglyceride content, and circulating non-esterified fatty acid concentrations in mice with HFD-induced obesity. Exenatide also enhanced SIRT1, AMPK, and acetyl-CoA carboxylase signalling, as well as fatty acid oxidation and mitochondrial biogenesis. SIRT1 deficiency or knockdown markedly attenuated these effects, suggesting that SIRT1 promotes a transition in white adipose tissue from lipid storage towards lipid mobilisation and oxidative utilization (Xu et al., 2016). Collectively, GLP-1RAs may alleviate obesity-related lipotoxicity and delay CKM progression by limiting excess lipid availability, promoting fatty acid oxidation, and attenuating the inflammation and fibrosis associated with ectopic lipid deposition.

#### 4.1.2 The impact on obesity-related kidney damage

In addition to reducing systemic fat load, GLP-1RAs may protect against obesity-related kidney injury by remodelling kidney lipid metabolism, preserving mitochondrial homeostasis, and improving kidney microvascular function. Wang et al. reported that liraglutide reduced kidney accumulation of fatty acids, cholesterol, and triglycerides; inhibited lipid uptake and synthesis; and enhanced fatty acid oxidation in rats HFD-induced obesity. These effects were accompanied by improvements in SIRT1–AMPK–PGC-1α signalling, the NAD^+^/NADH ratio, and ATP production, ultimately attenuating albuminuria, glomerulosclerosis, and interstitial fibrosis (Wang et al., 2018). Exenatide restored SIRT1 and PGC-1α expression, reduced mitochondrial ROS production and apoptosis, and mitigated lipotoxic injury by improving mitochondrial function in kidney tubular cells from HFD-fed mice and in palmitate-stimulated tubular epithelial cells (Wang et al., 2021).

GLP-1R agonists may also modulate obesity-related abnormalities in kidney haemodynamics. Reduced body weight and blood pressure lower the glomerular perfusion load, and GLP-1R signalling may also play a role in regulating sodium excretion and kidney vascular responses. Sukumaran et al. reported that 8 weeks of low-dose liraglutide treatment in high-salt-fed obese Zucker rats did not significantly alter body weight or blood glucose concentrations. Nevertheless, liraglutide upregulated kidney endothelial nitric oxide synthase (eNOS), improved kidney arteriolar dilation and blood flow, partially reduced albuminuria and glomerular dilatation, and attenuated the increase in blood pressure (Sukumaran et al., 2019). These findings suggest that improved kidney microvascular endothelial function may represent a kidney mechanism of GLP-1RA action distinct from its systemic metabolic effects.

Clinical studies provide corroborative support for these experimental findings. The SELECT trial enrolled 17,604 participants with CVD who were overweight or obese and did not have diabetes. The results showed that semaglutide educed the incidence of the composite kidney endpoint from 2.2% to 1.8%; after 104 weeks of treatment, the between-group difference in change in eGFR was 0.75 mL/min/1.73 m^2^ (Colhoun et al., 2024). Apperloo et al. enrolled 101 overweight or obese individuals with CKD without diabetes. After 24 weeks of once-weekly semaglutide 2.4 mg, the urinary albumin-to-creatinine ratio (UACR) was 52.1% lower than with placebo, accompanied by reductions in body weight and systolic blood pressure (Apperloo et al., 2025). In summary, available evidence suggests that GLP-1RAs may reduce the risk of obesity-driven progression of CKM from early adipose tissue dysfunction to intermediate and advanced stages characterised by CKM by reducing perikidney and intrakidney lipid burden, preserving kidney tubular mitochondrial homeostasis, and improving kidney microvascular function.

#### 4.1.3 The impact on obesity-related cardiac damage

GLP-1RAs may attenuate obesity-related cardiac injury by restoring metabolic coupling between adipose tissue and the myocardium (Akoumian et al., 2023). Reductions in visceral adiposity decrease excessive free fatty acid delivery to the heart, thereby limiting myocardial triglyceride deposition and the accumulation of lipotoxic intermediates, including ceramides and diacylglycerols (Bizino et al., 2020). Concurrent reductions in epicardial adipose tissue may lessen its mechanical constraint and pro-inflammatory paracrine effects on the adjacent myocardium and coronary arteries, thereby improving local lipotoxicity and coronary microvascular dysfunction. As myocardial lipid burden declines, metabolic flexibility in the utilisation of glucose and fatty acids may be restored, attenuating the reduction in energy efficiency associated with excessive fatty acid oxidation (Ma et al., 2024). Accordingly, the cardioprotective effects of GLP-1RAs in obesity may extend beyond reductions in systemic adiposity to include improvements in local myocardial metabolism and cardiac structure and function. Preclinical studies support this view. In a HFD-induced mouse model of obesity, Noyan-Ashraf et al. administered low-dose liraglutide for 1 week without inducing significant weight loss. Liraglutide improved cardiac function, restored the expression and distribution of desmolin 43 and endothelial nitric oxide synthase (eNOS), and reduced myocardial TNF-α concentrations, NF-κB activity, type I procollagen expression, and perivascular fibrosis (Noyan-Ashraf et al., 2013). These findings suggest that the cardioprotective effects of GLP-1RAs may arise from coordinated improvements in lipid metabolism, inflammation, intercellular junctions, and profibrotic signalling.

The STEP-HFpEF trial provided direct clinical evidence that semaglutide improves health status and functional capacity in obesity-related HFpEF. This randomised, double-blind, placebo-controlled trial enrolled 529 patients with obesity and HFpEF and compared once-weekly semaglutide 2.4 mg with placebo over 52 weeks. The Kansas City Cardiomyopathy Questionnaire Clinical Summary Score increased by 16.6 points with semaglutide and 8.7 points with placebo, yielding a between-group difference of 7.8 points. Six-minute walk distance increased by 21.5 m and 1.2 m, respectively, corresponding to a between-group difference of 20.3 m; body weight decreased by 13.3% and 2.6%, respectively (Kosiborod et al., 2023). Thus, semaglutide reduced body weight while improving HF symptoms and exercise capacity in patients with obesity-related HFpEF.

### 4.2 The effects of GLP-1 receptor agonists on glucotoxicity and associated cardiac and kidney damage

#### 4.2.1 Regulation of whole-body glucose homeostasis

GLP-1RAs promote insulin secretion in a glucose-dependent manner. When blood glucose concentrations rise, GLP-1R activation in pancreatic β-cells stimulates adenylate cyclase, increasing intracellular cAMP levels. This activates signalling pathways involving PKA and Epac2, which promote insulin granule recruitment and exocytosis. As blood glucose concentrations approach normal values, this insulinotropic effect diminishes; accordingly, GLP-1RA monotherapy carries a low risk of severe hypoglycaemia (Foll et al., 2023). Under hyperglycaemic conditions, GLP-1RAs also suppress inappropriate glucagon secretion, thereby reducing hepatic glycogenolysis and gluconeogenesis and lowering fasting and inter-meal glucose concentrations. Some GLP-1RAs additionally delay gastric emptying, slowing postprandial glucose entry into the circulation (Liu Q. K., 2024). Short-acting formulations, such as twice-daily exenatide, typically have a more pronounced effect on gastric emptying and postprandial blood glucose levels. However, with prolonged use of long-acting formulations, their effect on delaying gastric emptying may diminish due to rapid tolerance. Nevertheless, they provide more stable control of fasting and whole-day blood glucose levels (Jalleh et al., 2024). Beyond their pancreatic actions, GLP-1RAs may improve peripheral insulin sensitivity through weight loss and broader metabolic improvements. In a mouse model of diet-induced diabetes, Zhou et al. found that 14 days of liraglutide treatment significantly improved insulin sensitivity and increased AMPKα- and SIRT1-related signalling in the liver and adipose tissue, accompanied by enhanced fatty acid oxidation and reduced inflammation (Zhou et al., 2019). Collectively, these changes may reduce hepatic glucose output and improve insulin resistance in the liver, skeletal muscle, and adipose tissue.

Further clinical evidence supports these glycaemic effects. SUSTAIN 1 was a randomised, double-blind, placebo-controlled trial involving 388 treatment-naïve patients with T2DM who had inadequate glycaemic control despite diet and exercise. Participants received once-weekly semaglutide (0.5 mg or 1.0 mg) or placebo for 30 weeks. From a mean baseline HbA1c of 8.05%, HbA1c decreased by 1.45% and 1.55% with semaglutide 0.5 mg and 1.0 mg, respectively, compared with 0.02% with placebo. Semaglutide treatment was also associated with weight loss (Sorli et al., 2017). These findings indicate that semaglutide improves glycaemic control while reducing body weight in treatment-naïve patients with T2DM, thereby addressing two major components of metabolic burden.

#### 4.2.2 Effects on T2DM-related kidney damage

Building on improvements in systemic glucose metabolism, GLP-1RAs may attenuate T2DM-associated kidney injury by interrupting the progression from sustained hyperglycaemic exposure to glomerular filtration barrier dysfunction and tubulointerstitial remodelling. These agents enhance insulin secretion and suppress glucagon release in a glucose-dependent manner, thereby reducing hyperglycaemic exposure and glycaemic variability, which in turn limits the formation of AGEs and attenuates oxidative stress (Ojima et al., 2013). Preclinical studies further indicate that kidney GLP-1R signalling may suppress AGE–RAGE- and nicotinamide adenine dinucleotide phosphate oxidase (NOX)-related pathways, thereby reducing the production of ROS, asymmetric dimethylarginine and inflammatory mediators, preserving glomerular endothelial function, and alleviating podocyte injury and aberrant mesangial cell activation (Hendarto et al., 2012). Sourris et al. showed that GLP-1R deficiency increased kidney RAGE expression and aggravated kidney injury in diabetic mice, whereas concurrent RAGE deletion attenuated these changes. Conversely, liraglutide reduced kidney RAGE expression, suppressed myeloid progenitor cell proliferation, and promoted macrophage polarisation towards an M2-like phenotype, thereby reducing albuminuria and histological kidney injury (Sourris et al., 2024). GLP-1 RAs may also protect the glomerular filtration barrier and modulate regulated tubular cell death. Shen et al. reported that, in diabetic mice and high-glucose-stimulated kidney tubular epithelial cells, exendin-4 restored AMPK-related fatty acid metabolism and reduced iron accumulation, lipid peroxidation and tubular ferroptosis, accompanied by lower urinary albumin and kidney injury molecule-1 levels. Blocking macropinocytosis markedly attenuated these effects, suggesting that tubular uptake of exendin-4 may contribute to its cytoprotective action (Shen et al., 2024). Furthermore, in mice with HFD- and STZ-induced DKD, liraglutide attenuated podocyte injury and reduced the expression of NLRP3 inflammasome components and pyroptosis-related proteins, suggesting that the nephroprotective effects of GLP-1R agonists may also involve modulation of NLRP3-mediated pyroptosis (Shi et al., 2023). Collectively, these findings indicate that the kidney benefits of GLP-1 RAs may involve attenuation of inflammation, preservation of the filtration barrier, restoration of cellular energy metabolism and suppression of regulated cell death.

Further clinical evidence supports the kidney benefits of GLP-1 RAs, initially through prespecified kidney secondary outcomes in cardiovascular outcome trials. The LEADER trial enrolled 9,340 patients with T2DM at high cardiovascular risk. The composite kidney outcome occurred in 5.7% of patients receiving liraglutide versus 7.2% of those receiving placebo, with the benefit driven primarily by a lower incidence of new-onset persistent macroalbuminuria (Mann et al., 2017). Similarly, SUSTAIN-6 enrolled 3,297 patients with T2DM at high cardiovascular risk and showed that semaglutide reduced the incidence of new or worsening kidney disease from 6.1% to 3.8%, largely through a reduction in persistent macroalbuminuria (Marso et al., 2016). Within the CKM framework, these findings support a role for GLP-1 RAs in attenuating the progression from metabolic dysfunction to CKD and more advanced cardiovascular risk, particularly by reducing albuminuria and preserving kidney function.

#### 4.2.3 Effects on T2DM-related cardiac damage

Beyond reducing hyperglycaemic exposure, GLP-1RAs may attenuate T2DM-associated cardiac injury by reshaping myocardial substrate utilisation, improving coronary microvascular function and preserving cellular homeostasis (Karakasi et al., 2025). In the insulin-resistant myocardium, impaired glucose uptake and oxidation increase reliance on fatty acids for energy production. When fatty acid uptake exceeds mitochondrial oxidative capacity, lipid intermediates, including triacylglycerols, diacylglycerols, ceramides and acylcarnitines, accumulate within cardiomyocytes. This lipid overload can activate PKC–NOX signalling, increase ROS production and impair myocardial energetic efficiency (Ke et al., 2023). Preclinical studies have shown that liraglutide reduces myocardial lipid droplet accumulation and activates AMPK, thereby improving mitochondrial function and attenuating oxidative stress and myocardial fibrosis (Kuo et al., 2025). Liraglutide may also shift diabetic myocardial metabolism towards a more balanced pattern of substrate utilisation by enhancing pyruvate dehydrogenase activity and glucose oxidation (Sun et al., 2025).

Preclinical evidence further supports these mechanisms. Inoue et al. reported that, in streptozotocin-induced diabetic rats, 4 weeks of liraglutide treatment did not significantly alter blood glucose, insulin levels or body weight, but reduced myocardial accumulation of triglycerides and diacylglycerols. Liraglutide also downregulated fatty acid translocase and lipogenic proteins, suppressed PKC and NOX activity, and attenuated oxidative injury and cardiomyocyte apoptosis. These effects were accompanied by increased AMPK phosphorylation and SIRT1 expression, suggesting that the cardioprotective actions of liraglutide may be at least partly independent of its glucose-lowering effects (Inoue et al., 2015). Similarly, Exendin-4 reduced caspase-1 activity and the levels of pyroptosis-associated inflammatory mediators in diabetic rats and cardiomyocytes exposed to high glucose. These effects were associated with modulation of AMPK–TXNIP signalling, suggesting that suppression of cardiac pyroptosis may represent an additional mechanism underlying the protective effects of GLP-1RAs against diabetic myocardial injury (Wei et al., 2019). Clinical evidence further supports these cardiovascular benefits. The SOUL trial enrolled 9,650 patients with T2DM and concomitant atherosclerotic CVD and/or CKD. Oral semaglutide reduced the incidence of major adverse cardiovascular events from 13.8% to 12.0% and was associated with a 27% reduction in the risk of fatal or non-fatal myocardial infarction (McGuire et al., 2025). The STEP-HFpEF DM trial enrolled 616 patients with obesity, T2DM and HFpEF. After 52 weeks of semaglutide treatment, the Kansas City Cardiomyopathy Questionnaire clinical summary score improved by 7.3 points compared with placebo, while the 6-min walk distance increased by 14.3 m and C-reactive protein levels decreased by 42.0% (Kosiborod et al., 2024). Collectively, these findings indicate that GLP-1 receptor agonists may reduce atherosclerotic cardiovascular events while improving the obesity-related HFpEF phenotype in patients with T2DM. Within the CKM framework, these cardiovascular benefits may help attenuate progression from metabolic dysfunction to clinical CVD (Figure 3; Table 1).

**FIGURE 3:**
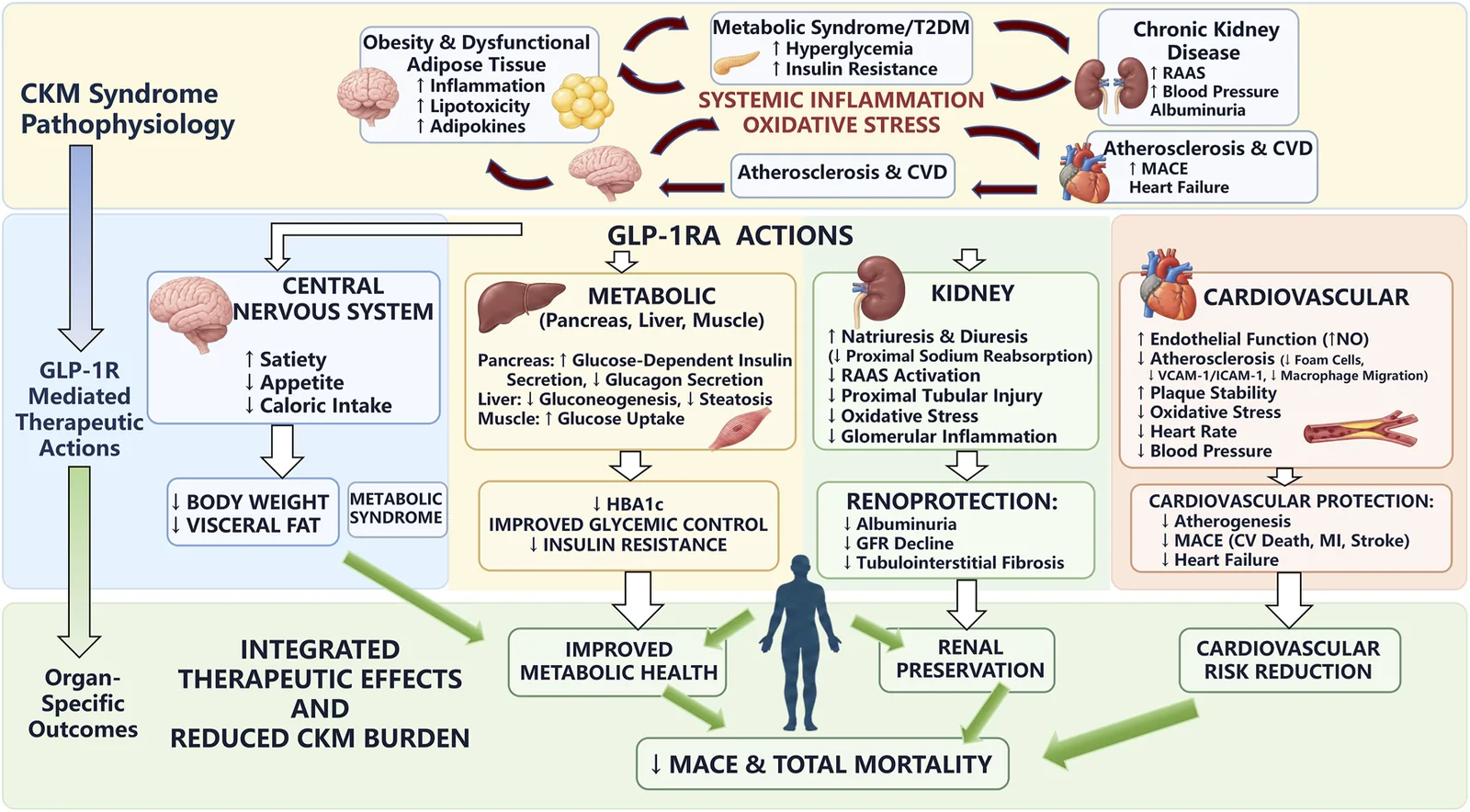
Mechanisms of GLP-1 receptor agonist-mediated metabolic, renal, and cardiovascular protection in CKM syndrome. GLP-1RAs exert coordinated metabolic and cardiorenal effects across multiple organs involved in CKM syndrome. Through central regulation of appetite and satiety, GLP-1RAs reduce energy intake, body weight, visceral adiposity, and ectopic lipid accumulation. Their metabolic actions further improve glucose homeostasis and insulin sensitivity by enhancing glucose-dependent insulin secretion and suppressing inappropriate glucagon release, thereby alleviating glucotoxicity and lipotoxicity. In the kidney, GLP-1RAs may improve renal haemodynamics and sodium handling while attenuating inflammation, oxidative stress, mitochondrial dysfunction, fibrosis, and tubular injury, contributing to reduced albuminuria and preservation of kidney function. In the cardiovascular system, improvements in endothelial function, vascular homeostasis, myocardial metabolism, inflammation, and fibrosis may contribute to reduced atherosclerotic cardiovascular events and improved cardiac function. Collectively, these integrated actions may interrupt the reciprocal interactions among metabolic dysfunction, kidney injury, and cardiovascular damage, thereby slowing CKM progression and reducing its overall disease burden.

**TABLE 1:** Representative clinical and basic studies of GLP-1–based therapy in CKM.

Condition	Study type	Intervention and dose	Sample size (n)	Duration	Target organ	Outcomes	References
Obesity	Clinical research	GLP-1 analogues (agent and dose varied by study)	45 studies (35 meta-analysed)	3–84 weeks	Adipose tissue Liver	↓TAT, SAT,VAT, EAT, LAT	(Akoumian et al., 2023)
Obesity	Clinical research	Semaglutide 2.4 mg s.c. once weekly vs. placebo	17,604 (8,803/8,801)	182 weeks	Kidney	↓5-component kidney outcome ↓eGFR ↓UACR	(Colhoun et al., 2024)
Obesity	Clinical research	Semaglutide 2.4 mg s.c. once weekly vs. placebo	101 (51/50)	24 weeks	Kidney	↓UACR ↓BW ↓SBP ↓eGFR	(Apperloo et al., 2025)
Obesity	Clinical research	Semaglutide 2.4 mg s.c. once weekly vs. placebo	529 (1:1)	52 weeks	Heart	↑KCCQ-CSS ↑6MWD ↓BW	(Kosiborod et al., 2023)
Obesity	Basic research	GLP-1^(7–36)^, 10 μg/kg Exendin-4, 250 ng/nodose ganglion	—	Acute (2–6 h)	Gut–liver vagal axis	↓TG, TRL-TG ↑portal–vagal GLP-1R signalling	(Hoffman et al., 2022)
Obesity	Basic research	Exenatide 24 nmol/kg i.p. once daily	—	8 weeks	Adipose tissue	↓BW, TG, FA ↑lipolysis, FAO, mitochondrial biogenesis ↑SIRT1,AMPK, ACC	(Xu et al., 2016)
Obesity	Basic research	Liraglutide 0.1 mg/kg s.c. twice daily	—	24 weeks	Kidney	↓Kidney FA, cholesterol, TG ↑ATP, NAD^+^/NADH ↓UACR, inflammation, glomerulosclerosis/fibrosis	(Wang et al., 2018)
Obesity	Basic research	Exenatide 0.12 mg/kg/day s.c.; 100 nM in HK-2 cells	—	6 weeks *in vivo* 24 h *in vitro*	Kidney Tubular cells	↑SIRT1, PGC-1α ↓ROS ↓tubular injury	(Wang et al., 2021)
Obesity	Basic research	Liraglutide 0.1 mg/kg/day s.c. by osmotic pump	—	8 weeks	Kidney	↑GFR, eNOS ↓SBP, albumin, ACR ↓glomerular enlargement, tubulointerstitial fibrosis	(Sukumaran et al., 2019)
Obesity	Basic research	Liraglutide 30 μg/kg s.c. twice daily; 100 nM *in vitro*	—	56 weeks	Heart	↓TNF-α,NF-κB ↓hypertrophy/fibrosis ↑eNOS/Cx43, AMPK, cardiac function	(Noyan-Ashraf et al., 2013)
T2DM	Clinical research	Semaglutide 0.5 or 1.0 mg s.c. once weekly vs. placebo	388 (2:2:1:1)	30 weeks	Systemic glucose metabolism	↓HbA1c, FPG ↓BW	(Sorli et al., 2017)
T2DM	Clinical research	Liraglutide up to 1.8 mg s.c. daily vs. placebo plus usual care	9,340 (4,668/4,672)	3.84 years	Kidney	↓Kidney composite ↓macroalbuminuria	(Mann et al., 2017)
T2DM	Clinical research	Semaglutide 0.5 or 1.0 mg s.c. once weekly vs. placebo	3,297 (1,648/1,649)	104 weeks	Kidney	↓new/worsening nephropathy ↓macroalbuminuria	(Marso et al., 2016)
T2DM	Clinical research	Oral semaglutide up to 14 mg daily vs. placebo	9,650 (4,825/4,825)	Mean 47.5 months median 49.5 months	Heart Kidney	↓MACE	(McGuire et al., 2025)
T2DM	Clinical research	Semaglutide 2.4 mg s.c. once weekly vs. placebo	310/306	52 weeks	Heart	↑KCCQ-CSS ↑6MWD ↓BW, CRP	(Kosiborod et al., 2024)
T2DM	Basic research	Liraglutide 0.2 mg/kg/day i.p	—	18 weeks	Adipose tissue	↑insulin sensitivity ↓BW, liver/perigonadal fat mass ↑p-AMPKα/SIRT1 ↓IL-1β/IL-6	(Zhou et al., 2019)
T2DM	Basic research	Liraglutide 50 μg/kg/day s.c. ± perindopril 2 mg/kg	—	20 weeks	Kidney	↓RAGE, NF-κB, MCP-1 ↑IL-10, M2 polarisation ↓AER	(Sourris et al., 2024)
T2DM	Basic research	Exendin-4 10 or 20 μg/kg/day s.c	—	8 weeks	Kidney	↑AMPK/FAO ↓lipogenesis/glycolysis ↓ lipid peroxidation, iron overload ↓KIM-1/UACR	(Shen et al., 2024)
T2DM	Basic research	Liraglutide 0.4 mg/kg s.c. twice weekly; 0.1 μM in H9c2 cells	—	24 weeks *in vivo* 24 h *in vitro*	Heart	↑AMPK, mitochondrial function ↓ROS, lipid droplets ↓myocardial fibrosis	(Kuo et al., 2025)
T2DM	Basic research	Liraglutide 0.3 mg/kg s.c. every 12 h	—	4 weeks	Heart	↑p-AMPK/SIRT1 ↓myocardial TG/DAG ↓PKC/NADPH oxidase ↓TUNEL/apoptosis	(Inoue et al., 2015)

### 4.3 Comparison and complementary roles of GLP-1RAs and SGLT2 inhibitors in CKM treatment

In addition to GLP-1RAs, SGLT2 is have become an important component of the comprehensive management of CKM. Although both drug classes provide cardiovascular and renal benefits beyond glycaemic control, their therapeutic profiles and clinical benefits differ.

Metabolically, GLP-1RAs improve glycaemic control and reduce body weight, visceral adiposity, and ectopic lipid deposition by enhancing glucose-dependent insulin secretion, suppressing inappropriate glucagon secretion, and reducing appetite and energy intake through central mechanisms. In contrast, SGLT2 inhibitors lower blood glucose by inhibiting glucose and sodium reabsorption in the proximal renal tubule, thereby promoting glucosuria and natriuresis. The resulting urinary energy loss contributes to modest reductions in body weight, while natriuresis and related haemodynamic effects contribute to lower blood pressure. Direct head-to-head studies of these two drug classes further illustrate their distinct metabolic profiles. The SUSTAIN 8 trial enrolled 788 patients with T2DM inadequately controlled with metformin. At 52 weeks, semaglutide 1 mg reduced HbA1c by 0.49 percentage points more than canagliflozin 300 mg and produced 1.06 kg greater weight loss (Lingvay et al., 2019). Similarly, in the PIONEER 2 trial, oral semaglutide achieved a greater reduction in HbA1c than empagliflozin at 26 weeks. At 52 weeks, the treatment policy estimand showed body-weight reductions of 4.7 kg with oral semaglutide and 3.8 kg with empagliflozin (Rodbard et al., 2019).

Renally, both drug classes confer protective effects, although through distinct mechanisms. SGLT2 inhibitors reduce sodium–glucose reabsorption in the proximal tubule and increase NaCl delivery to the macula densa, thereby restoring tubuloglomerular feedback and reducing diabetes-associated glomerular hyperfiltration and intraglomerular pressure. They may also protect the kidney by reducing tubular transport workload, improving energy metabolism, and alleviating renal hypoxia (Sen and Heerspink, 2021). Large-scale clinical trials have shown that the renal benefits of SGLT2 inhibitors extend beyond patients with diabetes. DAPA-CKD, which enrolled 4,304 patients with CKD, including those without diabetes, showed that dapagliflozin reduced the risk of the composite outcome of a sustained decline in eGFR of at least 50%, end-stage kidney disease, or death from renal or cardiovascular causes by 39% (Heerspin et al., 2020). Similarly, EMPA-KIDNEY, which enrolled a broader population of 6,609 patients with CKD, showed that empagliflozin reduced the risk of kidney disease progression or cardiovascular death by 28%, with consistent benefits in patients with and without diabetes (Herrington et al., 2023). In contrast, GLP-1RAs may confer renal protection through improvements in inflammation, oxidative stress, renal haemodynamics, and tubular cell homeostasis. Whereas earlier evidence for their renal benefits focused largely on reductions in albuminuria, the FLOW trial extended these findings to clinically important kidney outcomes. Among 3,533 patients with T2DM and CKD, semaglutide reduced the risk of the primary kidney composite outcome by 24% compared with placebo and slowed the annual decline in eGFR (Perkovic et al., 2024). Accordingly, within the CKD framework, SGLT2 inhibitors predominantly target the tubuloglomerular haemodynamic axis, whereas GLP-1RAs may address residual renal risk by mitigating upstream metabolic disturbances, including obesity, glucotoxicity, lipotoxicity, and inflammation. These complementary mechanisms provide a pathophysiological rationale for combining the two drug classes.

In terms of cardiovascular outcomes, the two classes of drugs have different areas of focus in terms of their clinical benefits. There is strong evidence that GLP-1RAs reduce the risk of ASCVD by promoting weight loss and improving glucose and lipid metabolism, reducing inflammation and improving vascular function. The SELECT trial enrolled 17,604 overweight or obese patients with a history of cardiovascular disease but without diabetes. Semaglutide 2.4 mg reduced the composite risk of cardiovascular death, non-fatal myocardial infarction or non-fatal stroke by 20 per cent, indicating that its cardiovascular benefits extend beyond its glucose-lowering effects (Lincoff et al., 2023). Conversely, SGLT2 inhibitors have demonstrated consistent benefits in patients with HF. Proposed mechanisms include natriuresis and reductions in plasma volume, preload, and afterload, together with improvements in cardiorenal haemodynamics and energy homeostasis. In EMPEROR-Preserved, which enrolled 5,988 patients with HF and a LVEF >40%, empagliflozin reduced the risk of the composite outcome of cardiovascular death or hospitalisation for HF by 21% compared with placebo, an effect driven primarily by fewer HF hospitalisations. Importantly, this benefit was consistent irrespective of diabetes status (Anker et al., 2021). The STEP-HFpEF DM trial further showed that semaglutide produced substantial weight loss while improving HF-related symptoms and exercise capacity in patients with obesity, T2DM, and HFpEF, a clinically relevant CKM phenotype (Kosiborod et al., 2024). From a cardiovascular perspective, SGLT2 inhibitors have robust evidence for reducing HF events, whereas GLP-1RAs reduce obesity-related metabolic burden and atherosclerotic cardiovascular events and may also improve the clinical phenotype of obesity-related HFpEF. Thus, the two drug classes show complementary profiles of cardiovascular benefit, addressing different components of cardiovascular risk across the CKM spectrum.

More importantly, emerging evidence supports the potential complementary use of these two drug classes in CKM. A 2024 meta-analysis published in Circulation, including 17,072 patients with T2DM, found that the effects of GLP-1RAs on MACE, hospitalisation for HF, and composite kidney outcomes were consistent irrespective of baseline SGLT2 inhibitor use. Conversely, analyses of SGLT2 inhibitor trials showed that their benefits on HF or cardiovascular death and CKD progression were similarly maintained irrespective of concomitant GLP-1RA use. Collectively, these findings suggest that the cardiorenal benefits of the two drug classes may be preserved when used together, supporting their potential therapeutic complementarity and the possibility of additive cardiorenal protection (Neuen et al., 2024; Apperloo et al., 2024). The 2026 AHA/ACC/ADA/ASN guideline for the management of CKM syndrome recommends individualising the use of GLP-1-based therapies, SGLT2is, or both according to the predominant CKM phenotype and comorbidities, including obesity, T2DM, CKD, ASCVD, and HF. SGLT2is are prioritised in patients with CKD or HF, whereas GLP-1-based therapies may provide additional benefit when obesity, substantial hyperglycaemia, or ASCVD risk predominates. In selected high-risk patients with T2DM, concomitant SGLT2i and GLP-1-based therapy may be considered to improve glycaemic control, CKM risk-factor management, and cardiovascular and renal outcomes (Panjrath et al., 2026; Ndumele et al., 2026b). Therefore, within the CKM continuum encompassing metabolic, kidney, and cardiovascular disease, GLP-1RAs and SGLT2 inhibitors are best viewed as complementary therapeutic strategies. By targeting distinct but interconnected aspects of metabolic dysfunction, kidney disease progression, and cardiovascular risk, these therapies support a shift from isolated risk-factor control towards integrated, multi-organ management of CKM.

### 4.4 The potential value of natural products interventions in the treatment of CKM and related cardiovascular and renal diseases

CKM encompasses interconnected disturbances in glucose and lipid metabolism, renal injury, and cardiovascular disease. Its onset and progression are driven by inflammation, oxidative stress, mitochondrial dysfunction, metabolic remodelling, and fibrosis. Both long-term clinical studies and experimental studies have demonstrated that natural products, including traditional Chinese medicines (TCM), have significant therapeutic effects on diverse diseases, such as metabolic disorders (Ndumele et al., 2026a; Chen et al., 2024; Yuan et al., 2025), cardiovascular disease (Lin H. et al., 2024; Ajibade et al., 2024; Ben-Azu et al., 2025), and kidney disease (Acquah-Mills et al., 2025; Dong et al., 2024; Wu et al., 2025; Mao et al., 2020; Chen et al., 2025; Miao et al., 2022). Accumulating evidence has demonstrated that metabolic disorders and renal injury could be improved by TCM prescriptions (Liu et al., 2026d; Li et al., 2024d; Amarasiri et al., 2025; Liu et al., 2026e; Ruan et al., 2024), herb extracts (Jiang et al., 2025; Gao et al., 2024; Gao et al., 2025; Wang et al., 2023b) and natural compounds (Miao and Vaziri, 2025; Li et al., 2024e; Wang Y. N. et al., 2024; Wang et al., 2025; Wang et al., 2026). Some natural products, including TCM, also show therapeutic potential for the treatment of CKM and cardiorenal syndrome (Wen et al., 2025; Tain and Hsu, 2024). Compared with interventions targeting a single risk factor or specific molecular target, natural products are characterised by their multi-component, multi-target, and multi-pathway regulatory properties, which may better align with the pathological complexity of CKM involving interactions among multiple organs and mechanisms. In recent years, related studies have gradually expanded from focusing on isolated metabolic abnormalities or single-organ injury to the integrated evaluation of metabolic, cardiovascular, and renal phenotypes, providing a basis for further exploring the potential application of natural products in the comprehensive management of CKM.

Tain et al. reviewed the links between polyphenolic natural products and cardiovascular, renal, and metabolic health within the CKM framework, suggesting that their antioxidant, anti-inflammatory, and metabolic effects may collectively modulate CKM-related phenotypes (Tain and Hsu, 2024). Similarly, Wen et al. used reverse network pharmacology to identify berberine from a natural-product library and established a CKM mouse model featuring metabolic dysfunction, diastolic cardiac dysfunction, and renal fibrosis through combined HFD and Nω-nitro-L-arginine methyl ester exposure. Research has found that berberine can improve abnormalities in glucose and lipid metabolism, cardiac diastolic function, and kidney damage. Its effects involve the synergistic regulation of pathways related to lipid metabolism, glucose metabolism, and fibrosis, suggesting that natural products may simultaneously intervene in metabolic, cardiac, and renal abnormalities associated with CKM through a multi-target mechanism (Wen et al., 2025). Fisetin is a naturally occurring flavonoid found in many fruits and vegetables. In diabetic low-density lipoprotein receptor-deficient mice with accelerated atherosclerosis, Zou et al. found that 12 weeks of fisetin treatment improved hyperglycaemia, glucose intolerance, and insulin resistance, while reducing aortic atherosclerotic lesions and attenuating renal dysfunction, glomerulosclerosis, and renal fibrosis (Zou et al., 2023). Oleuropein, a major phenolic compound in olive leaves and fruits, was found by Zheng et al. to reduce fasting blood glucose and improve glucose tolerance and insulin resistance after 15 weeks of continuous treatment in db/db mice with advanced T2DM (Zheng et al., 2021). Further studies of cardiac and renal complications in the same disease model showed that oleuropein attenuated mesangial expansion, renal inflammation, and fibrosis, while suppressing myocardial inflammation, collagen deposition, and apoptosis (Zheng S. et al., 2024).

Overall, research has gradually evolved from merely evaluating the hypoglycaemic and lipid-regulating effects of natural products to making comprehensive observations of multisystem phenotypes, such as cardiovascular and renal damage. In particular, certain natural products have demonstrated the potential to simultaneously improve metabolism and protect target organs in the CKM model or in composite disease models characterised by both metabolic abnormalities and concurrent cardiovascular and renal damage. The following section will further examine the two main disease pathways—early-stage obesity and T2DM—within the CKM framework. It will summarise research progress on the effects of natural products on related metabolic abnormalities and cardiovascular and renal damage, respectively. The aim is to explore candidate CKM intervention strategies with multisystem protective potential.

#### 4.4.1 Effects of natural products on obesity-related metabolic disorders and cardiorenal injury

Clinical studies on natural products targeting obesity-related CKMT phenotypes have so far focused primarily on metabolic abnormalities, while direct evidence of cardiac and renal damage remains largely confined to basic research. Koperska et al. conducted a randomised, double-blind, placebo-controlled trial involving 70 patients with metabolic dysfunction-associated fatty liver disease. The patients had no glucose metabolism abnormalities, but were overweight or had class I obesity. They were treated with 1,500 mg/day of berberine for 12 weeks. The results showed that the berberine group exhibited reductions in alanine transaminase levels and the De Ritis ratio, as well as demonstrating more favourable changes in total cholesterol, compared with the control group. This study suggests that berberine may improve certain obesity-related metabolic parameters (Koperska et al., 2024).

Regarding obesity-related kidney injury, Li et al. compared astragaloside IV with its water-soluble derivative, LS-102, in mice with HFD-induced obesity-related nephropathy. Both treatments attenuated perirenal adipocyte hypertrophy and macrophage infiltration and improved glomerular enlargement, mesangial matrix expansion, and renal interstitial fibrosis; LS-102 produced greater effects at the same dose (Li Z. et al., 2022). More recently, Xu et al. established a HFD-induced mouse model of obesity-associated glomerular disease and administered rhein, an anthraquinone derived from rhubarb (300 mg/kg/day), from week 12 for a further 12 weeks. Rhein reduced proteinuria and serum creatinine levels, alleviated glomerular lesions, and improved body weight, triglyceride concentrations, and inflammatory markers (Xu et al., 2025). These findings accord with the broader pharmacological profile of rhubarb. Miao et al. reported that rhubarb contains anthraquinones—including rhein, emodin, and chrysophanol—along with other bioactive constituents. Its reported activities include reno- and cardioprotection, anti-inflammatory and anti-fibrotic effects, and regulation of glucose metabolism. Emerging transcriptomic and metabolomic approaches are further clarifying the multi-component, multi-target action network of rhubarb, providing a basis for investigating rhubarb and its active constituents within the complex pathophysiology of CKM (Miao et al., 2025).

Regarding obesity-related cardiac injury, Yi et al. established a rat model of obesity-associated myocardial fibrosis through long-term high-fat feeding. Epigallocatechin gallate reduced selected blood lipid indices and creatine kinase-MB levels, decreased myocardial collagen deposition, and restored SCN5A expression (Yi et al., 2022). In HFD-induced obese mice, Wu et al. found that isoginkgetin, a biflavonoid from Ginkgo biloba, improved diastolic function, myocardial remodelling, and mitochondrial respiratory capacity without significantly reducing body weight or blood glucose. These effects were markedly attenuated by Nrf2 silencing (Wu et al., 2022). Overall, these findings suggest that certain natural products may target distinct obesity-driven CKM manifestations, including metabolic burden, renal injury, and myocardial remodelling. However, studies that concurrently assess all three domains and establish causal mechanisms remain limited.

#### 4.4.2 Effects of natural products on T2DM-associated metabolic, cardiac, and renal injury

Numerous clinical studies have examined natural products for the treatment of T2DM, although the available evidence has largely focused on glycaemic outcomes. The multicentre, randomised PREMOTE trial enrolled 409 patients with newly diagnosed T2DM and compared berberine, berberine plus probiotics, probiotics alone, and placebo over 12 weeks. HbA1c decreased by 0.99% in the berberine group, compared with 0.59% in the placebo group. Berberine-containing interventions also improved fasting and postprandial glucose levels and selected lipid parameters (Zhang et al., 2020). Yaikwawong et al. administered curcumin extract (1,500 mg/day) for 12 months to overweight or obese individuals with recently diagnosed T2DM receiving metformin. Treatment reduced HbA1c, fasting blood glucose, homeostatic model assessment of insulin resistance (HOMA-IR), body weight, and body mass index, while increasing homeostatic model assessment of β-cell function (HOMA-β) and adiponectin levels and reducing leptin levels (Yaikwawong et al., 2024). Together, these studies suggest that selected natural products may improve glycaemic control and insulin resistance when used alongside standard T2DM therapy.

Regarding T2DM-related kidney injury, Zhao et al. enrolled 413 patients with DKD and compared irbesartan, Huang Kui capsules, and their combination. Huang Kui capsules are a single-herb preparation derived from an ethanol extract of Abelmoschus manihot flowers. After 24 weeks, the combination regimen achieved a greater reduction in urinary albumin-to-creatinine ratio than irbesartan alone (Zhao et al., 2022). Preclinical studies have likewise provided experimental evidence that natural products can modulate renal injury. Li et al. screened a natural-compound library and identified bruceine A, a quassinoid isolated from the fruit of Brucea javanica. In db/db mice, 8 weeks of intraperitoneal bruceine A treatment (0.5 or 2.0 mg/kg) reduced UACR, serum creatinine, and blood urea nitrogen levels, while attenuating mesangial expansion, podocyte injury, and renal interstitial fibrosis. Mechanistically, bruceine A directly bound galectin-1, disrupting its interaction with receptor for activated C kinase 1 and suppressing high-glucose-induced inflammatory signalling in mesangial cells (Li H. et al., 2022).

Regarding diabetes-related myocardial injury, Jin et al. found that muxiangene lactone attenuated myocardial structural damage and fibrosis, improved cardiac function, and suppressed inflammation and oxidative stress in diabetic mice and high-glucose-exposed cardiomyocytes (Jin et al., 2023). In 2024, Zhang et al. established a mouse model of diabetic cardiomyopathy using a high-fat, high-sucrose diet combined with STZ administration. Gastrodin improved cardiac function and reduced myocardial fibrosis; subsequent cellular and intervention studies implicated the kallikrein-related peptidase 8 (KLK8)–protease-activated receptor 1 (PAR1) pathway in its anti-fibrotic effects (Zhang et al., 2024). These studies provide experimental support for using bioactive monomers derived from traditional Chinese medicine to ameliorate diabetes-related myocardial injury and remodelling. Research on natural products in T2DM has expanded beyond glucose metabolism and pancreatic function to encompass DKD and atherosclerosis. Some animal studies now concurrently assess cardiac and renal injury and their responses to intervention.

As CKM progresses from early metabolic abnormalities to intermediate and advanced stages of organ injury, renal and cardiovascular lesions increasingly interact rather than evolve independently. Kidney dysfunction may exacerbate cardiovascular injury through volume overload, activation of the RAAS and sympathetic nervous system, chronic inflammation, and uremic toxin accumulation. Conversely, cardiac dysfunction and venous congestion can impair renal perfusion and accelerate renal functional decline, creating a self-reinforcing cardiorenal cycle. Zhao et al. highlighted that cardiorenal syndrome involves haemodynamic disturbances and dysregulation of oxidative stress, inflammation, RAAS, Wnt/β-catenin, and TGF-β/Smad signalling. Their review summarised evidence that natural products and other complementary therapies may confer cardiac and renal protection by modulating these pathways (Zhao B. R. et al., 2025). As these pathological processes share a high degree of mechanistic overlap with cardiac and renal damage in the middle and late stages of CKD, CVD and cardio-renal syndrome, research into natural products in these conditions may provide further evidence for screening candidate intervention strategies for CKM with cross-organ protective potential. However, as the current evidence primarily stems from CKM’s constituent diseases or models of CKM, it cannot yet be equated with proof that natural products can improve overall clinical outcomes in CKM. Further validation is required in standardised CKM models and population-based studies categorised according to CKM stage.

#### 4.4.3 Complementary mechanisms of action between natural products and GLP-1 receptor agonists, and the potential for combined intervention

GLP-1RAs primarily act through GLP-1R signalling to regulate appetite, body weight, and pancreatic hormone secretion, thereby alleviating the upstream glucolipid metabolic burden of CKM. In contrast, natural products may exert multi-target effects on oxidative stress, inflammation, endoplasmic reticulum stress, mitochondrial dysfunction, and fibrosis. This potential mechanistic complementarity may therefore link improvements in metabolic homeostasis with broader modulation of cellular stress and target-organ injury. Clinical studies have shown that, in a randomised, double-blind, crossover trial involving 30 participants with hyperglycaemia and conducted by Cesar et al., a 12-week intervention involving an eriocitrin preparation resulted in a 17 per cent increase in GLP-1 levels. This was accompanied by reductions in blood glucose, insulin resistance, and levels of IL-6 and TNF-α (Cesar et al., 2022), suggesting that this natural product may influence both the incretin axis and inflammatory responses simultaneously. Basic research further supports this complementary mechanism. Gaballah et al. found that, in rats with T2DM, the combination of quercetin and liraglutide improved hyperglycaemia and insulin resistance more effectively than monotherapy alone, whilst also inhibiting XBP1/CHOP-mediated endoplasmic reticulum stress, oxidative stress and inflammation (Gaballah et al., 2017). Zhang et al. found that combined treatment with akebia saponin D and semaglutide attenuated glomerular injury and renal fibrosis in diabetic rats, with these effects associated with modulation of Klotho–p53 signalling (Zhang Q. et al., 2026). In addition, berberine metabolites, including berberrubine and palmatine, alleviated oxidative stress and mitochondrial dysfunction in intestinal L cells while promoting GLP-1 production and glucose-stimulated GLP-1 secretion (Yang W. L. et al., 2024). Collectively, these findings suggest that natural products may complement GLP-1RAs through multiple mechanisms, including enhancement of endogenous GLP-1 activity and modulation of inflammation, oxidative stress, and tissue remodelling.

## 5 Conclusion

CKM is more than the coexistence of obesity, T2DM, CKD, and CVD; it is a systemic disease continuum driven by persistent interactions among metabolic dysfunction, renal injury, and cardiovascular injury. Obesity and T2DM are key drivers of early CKM progression, with adipose tissue dysfunction, insulin resistance, hyperglycaemia, and ectopic lipid deposition collectively imposing sustained metabolic stress. Drawing on existing evidence, this review highlights several key mechanisms underlying CKM progression. First, glucotoxicity and lipotoxicity do not act in isolation but form a mutually reinforcing glucotoxic–lipotoxic network through insulin resistance, dysregulated lipid metabolism, and oxidative stress. Second, persistent metabolic stress disrupts mitochondrial function and energy-substrate utilisation and may promote renal tubular and cardiomyocyte injury through regulated cell-death pathways, including ferroptosis, pyroptosis, and necroptosis. Furthermore, beyond the canonical cAMP–PKA/Epac2 pathway, GLP-1/GLP-1R signalling may engage non-canonical, cell- and tissue-specific mechanisms in the heart and kidneys. Collectively, metabolic dysfunction, inflammation, oxidative stress, mitochondrial injury, and regulated cell death may establish cross-organ feedback loops among metabolic tissues, the kidneys, and the cardiovascular system. These interactions drive CKM from early metabolic abnormalities to structural cardiac and renal injury and, ultimately, overt cardiovascular disease.

Within this pathophysiological framework, the role of GLP-1RAs extends beyond glycaemic control. In addition to improving glycaemia, body weight, and adipose tissue function, GLP-1RAs may confer cardiorenal protection by mitigating glucolipotoxicity, preserving mitochondrial and energy-metabolic homeostasis, suppressing inflammation and oxidative stress, and modulating regulated cell death. SGLT2 inhibitors are likewise central to comprehensive CKM management, with established benefits in slowing CKD progression and reducing heart-failure risk. The two drug classes have complementary mechanisms and clinical benefits. Contemporary CKM management therefore emphasises selecting or combining therapies according to clinical phenotypes, including obesity, T2DM, CKD, atherosclerotic cardiovascular disease (ASCVD), and HF, shifting the focus from individual risk-factor control towards integrated reduction of metabolic, cardiovascular, and renal risk.

Alongside targeted therapies such as GLP-1RAs, the multi-component, multi-target, and multi-pathway properties of natural products may offer complementary avenues for comprehensive CKM intervention. Existing studies have examined their glucose- and lipid-regulating effects, improvements in insulin resistance, and attenuation of cardiac and renal injury across CKM-related phenotypes, including obesity-related kidney injury, myocardial remodelling, DKD, diabetic cardiomyopathy, and cardiorenal syndrome. In composite disease models, some natural products have concurrently improved glucose and lipid metabolism while mitigating cardiac and renal damage, suggesting multisystem regulatory potential. However, current evidence derives mainly from preclinical research and studies of individual CKM component diseases and is insufficient to establish improvements in overall clinical outcomes in CKM. These findings are therefore better regarded as a basis for identifying candidate natural products and potential CKM targets than as direct therapeutic evidence. CKM stages 0–4.

Several key issues in existing research remain to be addressed. Firstly, a composite animal model that can more accurately replicate the progressive evolution of needs to be developed. Currently, most animal studies focus on individual phenotypes, such as obesity, T2DM, CKD or CVD. Even when composite models are available, such as obesity combined with diabetes or diabetes combined with atherosclerosis, it is difficult to track the progression from metabolic abnormalities to structural and functional damage in the heart and kidneys within a single model. Future models could incorporate obesity and insulin resistance alongside T2DM, hypertension, renal injury, and atherosclerosis, while longitudinally assessing glucose and lipid metabolism, renal function, cardiac structure and function, and vascular lesions. Such models may better reproduce phenotypes corresponding to CKM stages 0–4 and, when integrated with single-cell, spatial-omics, and multi-omics approaches, enable analysis of cross-organ communication during disease progression. Second, a multi-omics biomarker framework is needed to stratify CKM stages 0–4 and predict disease progression. Current CKM staging relies primarily on conventional metabolic, renal, and cardiovascular indicators and may not fully capture biological heterogeneity or the risk of stage progression. Longitudinal cohorts integrating eGFR, UACR, cardiac injury biomarkers, imaging phenotypes, and proteomic, metabolomic, and transcriptomic data could identify candidate biomarkers for CKM stages and predict renal functional decline and cardiovascular events. Their incremental predictive value over established risk tools and generalisability across populations should then be validated. Third, the tissue- and cell-specific actions of GLP-1R require further elucidation to inform more precise targeting strategies. Beyond optimising long-acting and oral GLP-1RAs, future research should define GLP-1R expression, signalling bias, and tissue drug exposure across relevant cell types and explore tissue-selective or signalling-biased agonists. Such approaches may minimise off-target adverse effects while preserving metabolic and cardiorenal benefits and clarify the contribution of direct GLP-1R-mediated organ protection in CKM. Fourth, the effects of combined CKM intervention strategies should be systematically evaluated. Although GLP-1RAs and SGLT2 inhibitors have complementary mechanisms, evidence supporting combined natural-product and GLP-1RA therapy for CKM remains limited. Future studies should first establish the chemical composition, dose, and quality standards of candidate natural products. Using target-identification approaches and composite CKM models, rigorously designed monotherapy and combination-treatment studies should then evaluate glucose and lipid metabolism, renal function, cardiac structure and function, and safety to distinguish synergistic, additive, and independent effects. Stage-stratified clinical studies are subsequently needed to define the overall therapeutic value of these interventions in CKM.

Future CKM research should shift from single diseases, organs, and endpoints towards integrated studies of disease stage, cross-organ mechanisms, and individualised treatment responses. Experimental and clinical research frameworks that capture the natural trajectory of CKM are needed to characterise tissue-specific GLP-1R signalling, validate complementary therapeutic strategies, and develop multi-biomarker-based risk stratification. These efforts may clarify the appropriate target populations and treatment windows for interventions at different CKM stages and advance care from single-disease management towards stage-based, multisystem, precision interventions.

## Glossary

AGEs: Advanced glycation end products
AHA: American Heart Association
AMPK: AMP-activated protein kinase
ATP: adenosine triphosphate
BMI: body mass index
CKD: chronic kidney disease
CKM: cardiovascular-kidney-metabolic syndrome
CVD: cardiovascular disease
DKD: diabetic kidney disease
eGFR: estimated glomerular filtration rate
eNOS: endothelial nitric oxide synthase
Epac2: exchange protein directly activated by cAMP 2
GLP-1: glucagon-like peptide-1
GLP-1R: glucagon-like peptide-1 receptor
GLP-1RA: glucagon-like peptide-1 receptor agonist
Gs: stimulatory G protein
HF: heart failure
HFpEF: heart failure with preserved ejection fraction
NF-κB: nuclear factor-κB
NOX: nicotinamide adenine dinucleotide phosphate oxidase
PGC-1α: peroxisome proliferator-activated receptor gamma coactivator 1-alpha
PKA: protein kinase A
PKC: protein kinase C
RAAS: renin-angiotensin-aldosterone system
RAGE: receptor for advanced glycation end products
Rap1: Ras-related protein 1
ROS: reactive oxygen species
SIRT1: sirtuin 1
T2DM: type 2 diabetes mellitus
TGF-β: transforming growth factor-β
TNF-α: tumor necrosis factor-α
UACR: urinary albumin-to-creatinine ratio
